# Respiratory Safety Evaluation in Mice and Inhibition of Adenoviral Amplification in Human Bronchial Endothelial Cells Using a Novel Type of Chlorine Dioxide Gas Reactor

**DOI:** 10.3390/toxics10010038

**Published:** 2022-01-13

**Authors:** Hae-Sung Yang, Kyeong-Min Kim, Napissara Boonpraman, Sun-Mi Yoon, Jeong-Eun Seo, Min-Woo Park, Jong-Seok Moon, Su-Young Yoo, Sun-Shin Yi

**Affiliations:** 1 Department of Biomedical Laboratory Science, College of Medical Sciences, Soonchunhyang University, Asan 31538, Korea; comet7298@naver.com (H.-S.Y.); rudals6103@naver.com (K.-M.K.); eun4945@naver.com (J.-E.S.); 2 Department of Medical Science, Graduate School, Soonchunhyang University, Asan 31538, Korea; beam_napissara@hotmail.com (N.B.); sycshm0227@naver.com (S.-M.Y.); 3 Department of Integrated Biomedical Science, Soonchunhyang Institute of Medi-bio Science (SIMS), Soonchunhyang University, Cheonan 31151, Korea; pmw0269@sch.ac.kr (M.-W.P.); jongseok81@sch.ac.kr (J.-S.M.); 4 NON Inc., Geumsan 32713, Korea; magic8577@naver.com

**Keywords:** disinfectants, chlorine dioxide gas, Dr.CLO^TM^, adenovirus, single inhalation toxicity

## Abstract

Since the onset of the COVID-19 pandemic, there has been a growing demand for effective and safe disinfectants. A novel use of chlorine dioxide (ClO_2_) gas, which can satisfy such demand, has been reported. However, its efficacy and safety remain unclear. For the safe use of this gas, the stable release of specific concentrations is a must. A new type of ClO_2_ generator called Dr.CLO^TM^ has recently been introduced. This study aimed to investigate: (1) the effects of Dr.CLO^TM^ on inhibiting adenoviral amplification on human bronchial epithelial (HBE) cells; and (2) the acute inhalation safety of using Dr.CLO^TM^ in animal models. After infecting HBE cells with a recombinant adenovirus, the inhibitory power of Dr.CLO^TM^ on the virus was expressed as IFU/mL in comparison with the control group. The safety of ClO_2_ gas was indirectly predicted using mice by measuring single-dose inhalation toxicity in specially designed chambers. Dr.CLO^TM^ was found to evaporate in a very constant concentration range at 0–0.011 ppm/m^3^ for 42 days. In addition, 36–100% of adenoviral amplification was suppressed by Dr.CLO^TM^, depending on the conditions. The LC_50_ of ClO_2_ gas to mice was approximately 68 ppm for males and 141 ppm for females. Histopathological evaluation showed that the lungs of female mice were more resistant to the toxicity from higher ClO_2_ gas concentrations than those of male mice. Taken together, these results indicate that Dr.CLO^TM^ can be used to provide a safe indoor environment due to its technology that maintains the stable concentration and release of ClO_2_ gas, which could suppress viral amplification and may prevent viral infections.

## 1. Introduction

Recently, people have become very interested in environmental quarantine owing to the global pandemic of COVID-19. Moreover, many types of disinfectant have been introduced, including antibacterial and antiviral agents, due to an increase in time spent indoors [1,2,3,4]. Because most disinfectants come into direct contact with objects used daily, the complete removal of the disinfection agent is sometimes not achieved [5,6]. As a solution to these shortcomings, chlorine dioxide (ClO_2_) gas is a type of disinfectant that has recently attracted attention [7,8,9,10].

ClO_2_ gas is already known for its excellent effects on deodorization [11], sterilization, or inhibition of viral amplification [8,12,13,14]. It is safe and has eco-friendly properties, such as easy decomposition by light and cost-effectiveness. It can be used on most surfaces and a range of objects [14]. Therefore, ClO_2_ gas has been widely used for the disinfection of various medical apparatus. It can suppress the growth of various microorganisms in the surrounding environment without being applied directly to the object’s surface [8,13,15]. Thus, gas-type ClO_2_ can be used as an ideal disinfectant that can be applied in daily life at low concentrations. However, practically, its use has been limited due to technical difficulties in maintaining a constant concentration of ClO_2_ gas for evaporation [14]. In addition, there is a risk that the outflow of a high concentration of ClO_2_ gas may significantly irritate the human mucous membranes, particularly the respiratory alveolar epithelium [8,9]. Therefore, if a low concentration of ClO_2_ gas is maintained within a stable range for daily life, ClO_2_ gas can be used as an effective and safe quarantine material type from airborne microbes present in the surrounding environment [16,17,18,19]. Dr.CLO^TM^ (a stick type of NON, Inc.) is a product that can generate very low concentrations of ClO_2_ gas in an easy manner. It is sold in Korea and in over 35 countries, including the United States, Japan, and recently in China. Unlike other products, Dr.CLO^TM^ produces a constant concentration of ClO_2_ gas. It is supplied in a helpful form for individuals to use.

The use of ClO_2_ gas is still controversial in terms of safety [8,9,20,21,22]. Safety issues are constantly being raised, but many recent reports have stated that the effective use of ClO_2_ gas is beneficial in quarantine living environments and medical fields [7,10,12,16,18,23,24,25]. Most studies pointing out the dangers of ClO_2_ gas also warn of the risks of unintentional workplace accidents and unspecified hyperreactivity [20,21,22]. However, the use of ClO_2_ gas has already been tried in various fields, and a variety of methods for using ClO_2_ gas within the safe range can be tested at a time when continuous quarantine for a large proportion of indoor area is emphasized, for example in a pandemic situation such as COVID19.

Recently, many researchers have actively reported studies related to the safety and benefits of ClO_2_ gas using laboratory animals worldwide [1,7,8,9,15,16,18,21,23,24,25,26,27,28]. However, few studies have been conducted in Korea, because devices that can perform inhalation toxicity are currently not readily available [29,30]. Therefore, in this study, the histopathological evaluation (as a single-dose inhalation toxicity study) of the range of ClO_2_ gas concentrations produced by the ClO_2_ gas generator (Dr.CLO^TM^), which has been released to the market for multi-purpose use, was performed. Furthermore, a study was conducted on the histopathological changes in the respiratory system, particularly the lungs, at the various ClO_2_ concentrations created by Dr.CLO^TM^ of experimental animals (male and female ICR mice), following the direction of the Korean Ministry of Food and Drug Safety (MFDS) [31]. In addition, it was verified whether an anti-adenoviral effect occurred in human bronchial epithelial cells (HBE) from very low ClO_2_ gas concentrations.

## 2. Materials and Methods

### 2.1. Concentration and Duration of Chlorine Dioxide (ClO_2_) Gas Generation by Dr.CLO^TM^

Dr.CLO^TM^ was placed on the top of a sealed chamber (1 m × 1 m × 1 m; 1m^3^). A ClO_2_ gas measuring machine (PortaSens III D16 portable gas detector, Analytical Technology, Inc., Collegeville, PA, USA) was placed at the bottom of the chamber. The activated product was then measured at the same time every day for an hour at 28 °C for 42 days. Before the experiment, the chamber was ventilated. All measurement environments were maintained in the same way. The average value of the concentration measured five times was calculated. To provide a brief explanation of how Dr.CLO^TM^ maintains a low chlorine dioxide gas concentration over a long time, Dr.CLO^TM^ has a solid substance part with a glass ampoule part inside. Liquid substances are stored in the glass ampoules. Bending the Dr.CLO^TM^ as needed breaks the glass ampoule inside; the liquid substance meets the solid substance, causing a chemical reaction to occur, but this chemical reaction is carried out in very small amounts.

### 2.2. Measurement of Dissolved Cl^−^ Ions in Culture Medium

The following method was used to measure chloride ion concentration when ClO_2_ gas was dissolved in the cell culture medium for 24 h. A chloride assay kit (ab83372; Abcam Cambridge, UK) was utilized for measuring chloride ion concentration after 24 h from an activated Dr.CLO^TM^. In brief, one million cells were rapidly homogenized with 100 μL lysis buffer (pH 6.5–8.0). After centrifuging at 13,000 rpm for 10 min to remove insoluble materials, the supernatant was diluted with assay buffer. Samples (10~50 μL) were taken, and the well volume was adjusted to 50 μL with distilled water. Afterward, 150 μL of chloride reagent of was added to each well containing chloride standard or test samples. After incubation at room temperature for 15 min, optical density (OD) at 620 nm was measured using a microplate reader (Thermo Scientific Multiskan GO, Vantaa, Finland).

### 2.3. Adenoviral Infectivity Titer

#### 2.3.1. Cell Culture

Human Beas-2B bronchial epithelial cells (CRL-9609^TM^, ATCC, Manassas, VA, USA) were cultured in DMEM (Invitrogen, Life Technologies, Grand Island, NY, USA) supplemented with 10% (*v*/*v*) heat-inactivated FBS, 100 units/mL penicillin, and 100 mg/mL streptomycin.

#### 2.3.2. Viral Forming Units (Titer)

For rapid measurement of the infected viral forming units in adenoviral-infected human bronchiolar epithelial cells, a QuickTiter^TM^ Adenovirus Titer Immunoassay kit (VPK-109) from Cell Biolabs, Inc. (San Diego, CA, USA) was used. Instead of using HEK-293 cell line provided by default in this kit, we used the human bronchial epithelial (HBE) cell mentioned earlier to determine the effect of ClO_2_ gas on the respiratory epithelium. In brief, immediately before recombinant adenoviral (Ad-β gal) infection, a 10-fold serial dilution of viral sample from 10^−3^ to 10^−7^ was performed. HBE cells were harvested and resuspended for viral infection in culture medium at 5 × 10^5^ cells/mL. After dispensing 1 mL of the cell-resuspended culture medium in a 12-well plate and incubating at 37 °C with 5% CO_2_ for an hour, ten-fold diluted viral samples were prepared in culture medium. Then, 100 μL of the diluted viral sample was dropwise added to each well of a 12-well assay plate. Infected cells were incubated at 37 °C with 5% CO_2_ for two days. At this time, Dr.CLO^TM^, after being fully activated for 48 h, was placed into the incubator containing test groups. Two days later, culture medium was were slowly removed from wells. Infected HBE cells were fixed by gently adding 0.5 mL cold methanol down the side of each well of the 12-well assay plate and then incubating the plate at −20 °C for 20 min. Afterward, immunostaining was performed using 1× anti-hexon primary antibody solution, 1× HRP-conjugated secondary antibody, and 1× DAB solution. Viral titers (infectious units/mL) were calculated as the average number of positive cells per well using the following equation:

$$\mathrm{Viral}\;\mathrm{titer}\;\left(\mathrm{ifu}/\mathrm{mL}\right)=\frac{\mathrm{Average}\;\mathrm{positive}\;\mathrm{cells}\;\left(\mathrm{per}\;\mathrm{field}\right)\times \left(\mathrm{Dilution}\;\mathrm{factor}\right)}{\left(0.1\;\mathrm{mL}\right)}\;$$

#### 2.3.3. Inhibition of Functional Titer Virus Infectivity by ELISA

A QuickTiter^TM^ Adenovirus titer ELISA Kit (VPK-110; San Diego, CA, USA) and an antibody against adenovirus hexon proteins were used to quantitate infected cells to measure the reduction in recombinant adenoviral infection in the HBE cell line by Dr.CLO^TM^. This assay was performed according to the manufacturer’s instructions. In brief, immediately before recombinant adenoviral (Ad-β gal) infection, a 2-fold serial dilution of Ad-β gal positive control was prepared in culture medium. Firstly, the viral stock was diluted at 1:2000, and eight sterile tubes were labeled as #1 to #8. Then, 500 μL of the 1:2000 diluted Ad-β gal viral sample was added to tube #1 and mixed well. Subsequently, 500 μL of diluent from tube #1 was transferred to the next tube. These steps were repeated for each tube, through to tube #7. Tube #8 was used as a blank. For accurate assessment of adenoviral titer, one of these dilutions for an unknown viral sample was to be within the range of the Ad-β gal standard curve (4.0 × 10^3^ IFU/mL to 2.5 × 10^5^ IFU/mL). For adenoviral infection, HBE cells were harvested and resuspended in culture medium at 5 × 10^5^ cells/mL, with 100 µL seeded into each well of a 96-well plate and incubated at 37 °C, 5% CO_2_ for an hour. Serial dilutions of the Ad-β gal positive control and viral samples in culture medium were prepared. A diluted viral sample (50 μL) was then dropwise added to each well of a 96-well assay plate. Infected cells were incubated at 37 °C with 5% CO_2_ for two days. At this time, a Dr.CLO^TM^, after being fully activated for 48 h, was placed into the incubator containing test groups. Two days later, culture medium was slowly removed from wells. Infected HBE cells were then fixed by gently adding 100 μL of cold methanol down from the wall of each well of the 96-well assay plate and incubating the plate at −20 °C for 20 min. Afterward, immunoassay was performed using 1× anti-hexon primary antibody solution and 1× HRP-conjugated secondary antibody. The reaction was developed with TMB substrate solution for 5 to 10 min. Finally, the reaction was stopped by adding 100 μL Stop solution to each well. The optical density of each well was then measured at 450 nm on a 96-well plate reader. The viral titer was calculated based on the standard curve from Ad-β positive control titrations.

### 2.4. Animal Preparation

Seventy 8-week-old ICR(CD1) male and female mice, respectively, were used in this study (140 animals in total). ICR mice are common experimental animals since they have no particular disease, pathogen, or genetic defects. They have been widely used in inhalation toxicity studies [32,33,34,35]. These animals were purchased from Oriental Bio (Seong-nam, Korea) and used for single whole-body ClO_2_ exposures in this study. Mice were housed at room temperature (22 ± 3 °C) and 30~70% humidity under a 12 h light:dark cycle from 07:00 to 19:00. Both males and females were divided into six groups based on the ClO_2_ gas concentration range and a ClO_2_ gas-untreated group. Ten animals were allocated to each group. These animals were given free access to a normal chow diet (2018S; Teklad diet, Envigo, Indianapolis, IN, USA) and water unless indicated otherwise for experiments. The Soonchunhyang University Institutional Animal Care and Use Committee (IACUC) approved all experiments and procedures (Approval number: SCH20_0033).

### 2.5. Study Designs for Single ClO_2_ Gas Whole-Body Exposure in Chambers

In general, a specially designed chamber device is required to achieve direct exposure of an animal’s respiratory system to the gas. For this study, the chamber was custom-made with the following functional design by Jeongdo Bio & Plant Inc. (Seoul, Korea). The ventilated animal chamber system was composed of six chambers (245 × 395 × 250 mm each) for ClO_2_ gas inhalation. The specifics of each chamber were as follows:

- Material: acryl;
- Floor: SUS304 stainless steel;
- Air in & out ventilation count: 12 times/h.

Since a single Dr.CLO^TM^ generates a very low concentration of ClO_2_ in the chamber, it was necessary to activate several Dr.CLO^TM^ sticks to create a detectable ClO_2_ concentration by the detector. Multiple sticks of Dr.CLO^TM^ were activated to generate ClO_2_ gas. The gas concentration was measured at regular time intervals (every 30 min) using a ClO_2_ detector (Porta Sens Gas Leak Detector (Model C16; Analytical Technology, Inc., Collegeville, PA, USA).

After waiting until a specific concentration was reached and stable (48 h), many Dr.CLO^TM^ devices were located in the chamber. Gas concentration maintenance of the chambers’ ventilation was then performed. Optimization of maintaining a particular concentration of chlorine dioxide gas for the number of Dr.CLO^TM^ was conducted.

#### 2.5.1. Maintaining ClO_2_ Gas Environment during the Study

Dr.CLO^TM^, which was activated 48 h before the experiment, was placed in every chamber for each concentration to maintain the corresponding concentration. The maximum number of ventilations in the chamber was 12 times/h. Once every 30 min, a ClO_2_ detector (Porta Sens Gas Leak Detector Model C16; Analytical Technology, Inc., Collegeville, PA, USA) was used to check whether the concentration was maintained for each chamber. The concentration was kept constant for 6 h. Since ClO_2_ gas is easily decomposed by light and air is introduced and exhausted into the chamber, ClO_2_ gas may not be constantly maintained at the specified concentration. Thus, pre-activated Dr.CLO^TM^ sticks were to be added or removed if necessary to maintain the concentration of ClO_2_ gas within a predetermined range (Table 1). After inhaling gas for 6 h in specially designed chambers, animals were observed for 14 days for changes in behavior and survival, according to the guidelines of the “Single dose inhalation toxicity test” provided by the Ministry of Korean Food and Drug Safety (MFDS) (Appendix 10) [31].

#### 2.5.2. Groups for Single ClO_2_ Gas Exposure in Chambers

Since no animal deaths occurred at low concentrations of ClO_2_ gas, the concentration range should be set somewhat higher to obtain LC_50_ of ClO_2_ gas. In other words, this study is a single-dose inhalation toxicity study and should be the basis for subsequently chronic repeated inhalation toxicity study. Therefore, a concentration gradient that can obtain not only LC_50_ but also LC_10_ must be established. Animals were less likely to die at moderate concentrations of chlorine dioxide, so an extremely high concentration of chlorine dioxide had to be set, as shown in this study. Before carrying out this study, data on animals’ reactions to chlorine dioxide gas were collected through preliminary research. The concentration range was determined considering the scope of our chlorine dioxide gas meter. Therefore, as shown in Table 1 below, male and female mice were allocated to six concentration groups (ten animals in each group), respectively. Several Dr.CLO^TM^ sticks (portable ClO_2_ gas generators) were placed in each chamber.

### 2.6. Histopathologic Evaluation and Scoring of Lung Alveolar Epithelium

Immediately after organ removal from mice, lung tissue samples were fixed in 10% neutral-buffered formalin, dehydrated in graded concentrations of ethyl alcohol, cleared in xylene, and embedded in paraffin. Embedded tissues were sectioned to 4 μm in thickness. Obtained sections were stained hematoxylin and eosin and Masson’s trichrome. Slides were examined with an automated Olympus (BX53) system to capture ultra-high-resolution images. DP80 with Olympus stream (Olympus, Waltham, MA, USA) images were captured. All histopathological changes in each lung tissue were evaluated, including intra-alveolar hemorrhage, alveolar cell edema, alveolar wall disruption, alveolar congestion with protein-rich fluid, and leukocyte infiltration. They were scored on a scale from 0 to 3 (Table 2) [36,37,38]. The average of these scores was used for comparison.

### 2.7. Statistics

All data are presented as mean ± SD or SEM. All statistical tests were analyzed using a Mann–Whitney U test (non-parametric) performed using a statistical software package (GraphPad Prism version 9.0, GraphPad Software Inc. (San Diego, CA, USA)) for comparison of multiple groups. *p* values less than 0.05 were considered statistically significant.

## 3. Results

### 3.1. ClO_2_ Gas Concentrations and Duration by Dr.CLO^TM^

**Figure 1:**
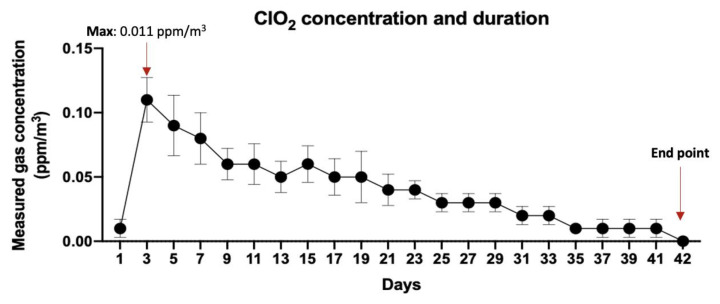
Safe duration and concentration of ClO_2_ gas controlled by Dr.CLO^TM^. After activation of Dr.CLO^TM^, the maximum concentration of ClO_2_ gas was 0.011 ppm/m^3^. It was confirmed that the concentration was stably maintained between 0.000 and 0.011 ppm/m^3^ for 42 days after Dr.CLO^TM^ activation. Error bars represent mean ± SD.

The change in the concentration of ClO_2_ gas maintained in the unit space (m^3^) when one Dr.CLO^TM^ is activated is shown. It was observed that chlorine dioxide gas was maintained at an almost constant concentration for 42 days, between 0 to 0.011 ppm. After activation of Dr.CLO^TM^, it was confirmed that a relatively low concentration of chlorine dioxide gas was continuously and constantly maintained (Figure 1). There was no temporary increase in the concentration of chlorine dioxide gas.

The maximum concentration exerted by Dr.CLO^TM^ was recorded as 0.011 ppm/m^3^, and the concentration gradually decreased with time. The presence of ClO_2_ is confirmed on the 41st day, but almost all values converged to 0 on the 42nd day, indicating loss of activity.

### 3.2. Dissolved Chloride Concentration in Culture Medium

**Figure 2:**
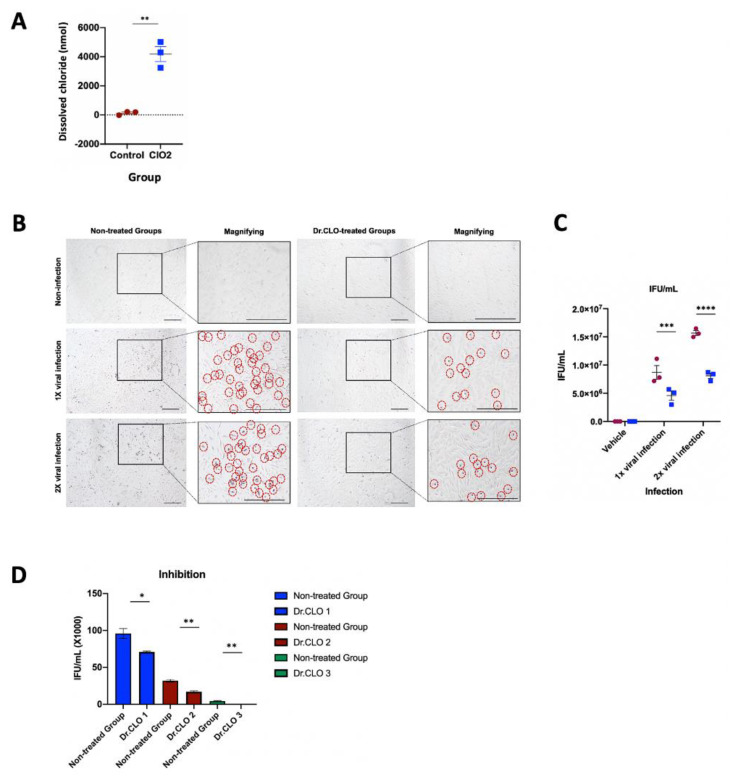
Quantitative and qualitative inhibitory effect of treatment by Dr.CLO^TM^ on adenoviral amplification. (**A**) The dissolved chlorine concentration in cell culture medium after activation of Dr.CLO^TM^ for 24 h. (**B**) When human bronchial epithelial (HBE) cells were inoculated with multiple adenovirus doses (1× & 2×) using the immunocytochemistry (ICC) method, infected positive cells showed purple reactions. Infected cells are marked with red dotted circles. (**C**) Results of positive cells not treated with Dr.CLO^TM^ and the number of positive cells inhibited when treated with Dr.CLO^TM^. Dr.CLO^TM^ showed inhibitory effects on both 1× and 2× viral infections. (**D**) Adenoviral titers (IFUs/mL) inoculated randomly at three different concentrations were significantly reduced by Dr.CLO^TM^. The black square box represents the enlarged cell shape. The error bars indicate mean ± SEM; *, *p <* 0.05; **, *p <* 0.005; ***, *p <* 0.0005; ****, *p <* 0.0001.

When one Dr.CLO^TM^ standard stick (a portable ClO_2_ gas generator) was incubated for 24 h in an incubator with a volume of 0.15 m^3^ with a plate in which cells and medium were seeded, the concentration of chlorine after the gas injected by Dr.CLO^TM^ was dissolved in the culture medium was measured. Dissolved chlorine concentrations in the medium were calculated using the kit. Results were: (1) non-treated group, 133.3 ± 74.05 nmol/mL, and (2) Dr.CLO^TM^ treated group, 4186.0 ± 513.3 nmol/mL (Figure 2A).

### 3.3. Calculation of Adenoviral Infection Titer (Infectious Unitw/mL) in Human Respiratory Epithelial Cells

This result is calculated based on the quantitation of naphthol-positive cells and virus-infected cells determined by immunocytochemistry (Figure 2B).

By varying the virus inoculation random titer (1× & 2×), it was possible to calculate the number of inoculated virus particles, indicating the change in IFU according to the virus inoculation amount: (1) 1× adenoviral inoculation, (8.7 ± 0.25) × 10^6^ IFU/mL; (2) 2× adenoviral inoculation, (15.7 ± 0.53) × 10^6^ IFU/mL (Figure 2C).

After inoculating adenovirus into human respiratory epithelial cells in a 12-well plate (triplicate) at different inoculation doses (1× & 2×), it was then determined how much virus infection was suppressed by treatment with Dr.CLO^TM^ for 24 h. The results are as follows: (1) suppressed infectivity in an infectious environment after 1× viral infection, (4.6 ± 0.21) × 10^6^ IFU/mL; (2) suppressed infectivity in an infectious environment of 2× viral infection, (8.1 ± 0.33) × 10^6^ IFU/mL (Figure 2C). Viral amplification in wells treated with Dr.CLO^TM^ for 24 h was inhibited by 52.9% for the 1× adenovirus inoculation group, and 51.6% for the 2× adenovirus inoculation group, compared to the non-treated group (Figure 2C).

### 3.4. Inhibition of Adenoviral Infection (ELISA Method)

When three random different virus concentrations were inoculated and Dr.CLO^TM^ treatment was used for 24 h, the decrease in virus amplification was measured by ELISA. A standard curve was obtained according to virus concentration and the amount of virus reduction by Dr.CLO^TM^ was measured. As the results, 36% reduction for the first random inoculum [(95.8 ± 6.70) × 10^3^ IFU/mL ⇨ (70.9 ± 1.20) × 10^3^ IFU/mL], 47.1% reduction for the second random inoculum [(32.0 ± 1.73) × 10^3^ IFU/mL ⇨ (17.0 ± 1.20) × 10^3^ IFU/mL], and 100% reduction for the third random inoculum [(4.3 ± 0.55) × 10^3^ IFU/mL ⇨ (0.0 ± 0.00) × 10^3^ IFU/mL] were obtained (Figure 2D). The above results confirmed that the infectivity of adenovirus was significantly reduced by ClO_2_ gas generated by Dr.CLO^TM^ into the cell culture medium.

### 3.5. Animal Body Weight Changes

**Figure 3:**
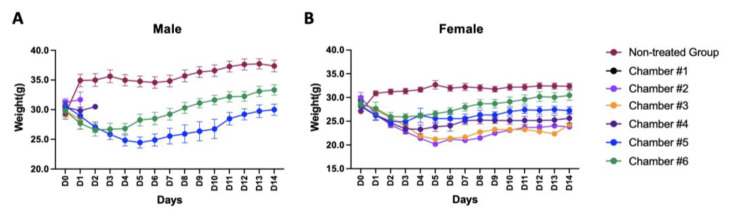
Body weight changes during the experiment. (**A**) For male animals, high concentrations of ClO_2_ gas (Chamber #1–Chamber #4) resulted in high mortality. However, mice in Chamber #5 and Chamber #6 recovered their body weight gradually from D4–5. (**B**) For female animals, all animals in Chamber #1 died after exposure to a concentration of 240 ppm or more. However, animals corresponding to the remaining concentrations were resistant to ClO_2_ gas and survived compared to males. Error bars represent mean ± SEM.

Seven concentration groups, including an untreated group, were used for exposure for 6 h, and the body weight changes were then observed for 14 days. We set very high gas concentrations that could not be reached in real life to obtain the LC_50_ for respiratory toxicity by Dr.CLO^TM^. The changes in the body weight of male and female animals were observed; the results are shown in Figure 3. Extreme body weight changes were detected at specific concentrations during the 14-day observation period. As shown in the graph, a significant decrease in body weight was initially observed at all gas concentrations. However, a tendency for the recovery of body weight was found six days after exposure in the surviving animals in most groups (Figure 3).

### 3.6. Animal Death after High Concentrations of Single ClO_2_ Gas Inhalation to Determine LC_50_

After inhaling gas for 6 h in specially designed chambers, the animals were observed for changes in behavior and survival for 14 days, according to the guidelines of the “Single dose inhalation toxicity study (Appendix 10)” in Standard for Toxicity Study of Pharmaceuticals provided by the MFDS [31]. Since there was a significant difference in survival rates of males and females over 14 days according to the results (Table 3), there was a difference in LC_50_ value for chlorine dioxide gas between males and females. The LC_50_ was approximately 68 ppm for males and 141 ppm for females. The LC_10_ was approximately 24 ppm for males and 30 ppm for females. Since the concentration of gas could not be specified, the LC_50_ was calculated using the median value of the concentration of each group.

### 3.7. Histopathological Findings

#### 3.7.1. H&E Staining and Masson’s Trichrome Staining

**Figure 4:**
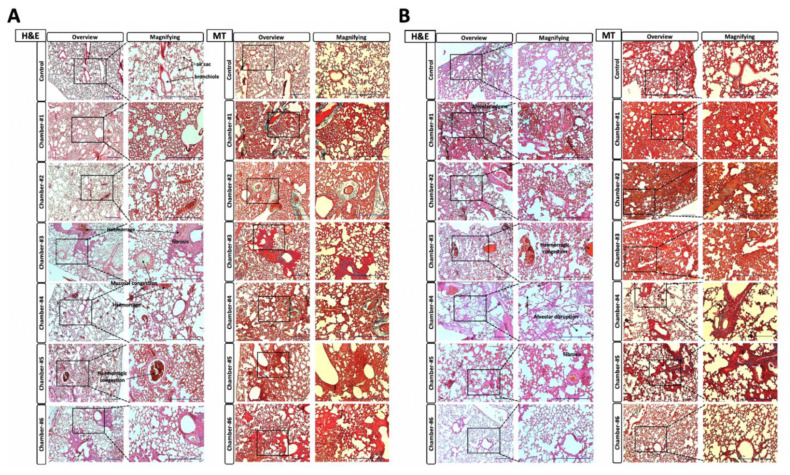
Lung tissue hematoxylin & eosin (H&E) and Masson’s trichrome (MT) staining results for each group. (**A**) Male histopathological staining images according to ClO_2_ gas concentration. From Chamber #1 to Chamber #5, edema in alveolar cells increased fibrosis of interstitial tissue. Mucosal cohesion and thromboembolic infraction of the alveolar lumen are recognized. (**B**) Histopathological staining images of female mice by ClO_2_ gas concentrations. From Chamber #1 to Chamber #5, edema in alveolar cells increased fibrosis of interstitial tissue. Findings of mucosal congestions and thromboembolic infarction in the alveolar lumen are similar to those in males. However, compared to males, alveolar lumen accounted for a higher proportion. Distraction of the alveolar wall was recognized. Black square boxes are enlarged parts of lesions. Control: non-treated group; Chamber #1: 240 ppm < group; Chamber #2: 200~240 ppm group; Chamber #3: 150~200 ppm group; Chamber #4: 100~150 ppm group; Chamber #5: 50~100 ppm group; Chamber #6: 20~50 ppm group. H&E = hematoxylin and eosin; MT = Masson’s trichrome. Scale bar = 100 μm.

Histopathological evaluation of the lung lobes of surviving animals after exposure to high concentrations of chlorine dioxide was performed (Figure 4). Through hematoxylin and eosin (H&E) and Masson’s trichrome (MT) staining, histopathological changes were observed in animals recovering in 14 days. Concentration-dependent changes were observed. In addition to an increase in connective tissues around the alveoli, thickening of the alveolar wall, inflammatory cells in the alveoli, congestion, bleeding of alveoli and blood vessels by fibrin or fibrin networks, and the abnormal disruption of the alveolar wall were recognized. In both males and females, relatively large morphological abnormalities were not observed in the group treated with the gas in the range of 20–50 ppm. However, the alveolar walls of animals exposed to ClO_2_ gas concentrations at 100–150 ppm were significantly thickened. This indicated that ClO_2_ gas at high concentrations could affect CO_2_–O_2_ gas exchange in the alveoli.

#### 3.7.2. Histopathological Examination Scoring for Lung

Based on the scoring results from histological evaluation of extremely high concentrations of ClO_2_ gas exposure, neither males nor females differed significantly from the control group at a Dr.CLO^TM^ concentration of 20–50 ppm. In the case of females, even at 50–100 ppm higher than males, there was no significant difference (Table 4 and Table 5). 

#### 3.7.3. Area Comparison of the Alveolar Air Sac

**Figure 5:**
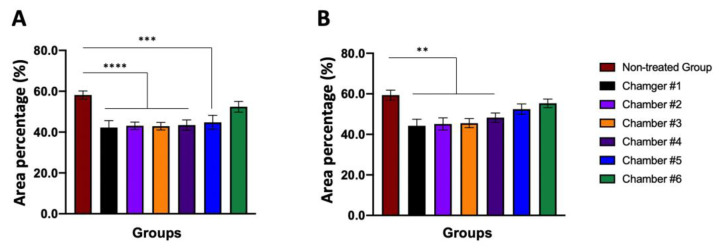
Graph showing the space of the alveolar sac lumen within the lung lobe. The area ratio of the space where gas exchange can occur per unit area is shown. (**A**) When comparing results in males, each concentration in Chamber #1–Chamber #5 shows a significant difference for ClO_2_ gas compared to the non-treated group. (**B**) When comparing results in females, each concentration in Chamber #1–Chamber #4 resulted in a significant difference for ClO_2_ gas compared to the non-treated group. **, *p* < 0.005; ***, *p* < 0.0005; ****, *p* < 0.0001. Error bars represent mean ± SEM.

Stimulation with chlorine dioxide gas caused congestion, bleeding, edema, and blockage of the alveolar cavity by secretions, which could significantly reduce the area occupied by the alveolar cavity compared to normal animals (Table 6). On the other hand, the percentage (%) occupied by the alveolar cavity in the observation screen is shown as a graph (Figure 5).

## 4. Discussion

In recent years, threatening viruses such as SARS-CoV, MERS-CoV, and SARS-CoV-2 (COVID-19) have become a global concern; further, we are entering an era where we do not know what types of epidemic will arise in the future [23,39]. In addition, reports of mass infections and zoonoses caused by various microorganisms that have become indigenous and a public health problem in each region are increasing [40,41]. To secure safety from these threatening microorganisms, we think that having a safe and convenient disinfection form for indoor environments and personal hygiene is a critical strategy. Furthermore, it is expected that the demand for effective disinfectants after the COVID-19 pandemic era will increase. For this reason, we believe that it is necessary to pay attention to the utilization and scalability of ClO_2_ gas among various disinfectants. ClO_2_ gas does not generate cancer-causing trihalomethanes [42,43]; the disinfecting effect of ClO_2_ is less affected by pH than chlorine, it has a less irritating odor, and has excellent concentration stability during storage [43]. Nevertheless, consumers are reluctant to use ClO_2_ gas due to the dangers of high concentrations of ClO_2_ gas [5,9]. However, if there was technology that could can efficiently control the concentration of this gas, it could be used for various purposes, such as grain storage [44,45], food storage through inhibition of microbial growth [46,47,48,49], disinfection of large buildings [50,51,52], and veterinary [53,54] or medical [8] use. As such, there is already widespread use of ClO_2_, but there are still concerns that it may be dangerous for infants [34,55], the elderly [34], and people with sensitive respiratory systems [34]. According to some studies, exposure to 50 ppm ClO_2_ gas for a short period is very dangerous and exposure at 2000 ppm can lead to death [20,21,56]. Pulmonary and hemodynamic changes were observed when an inhalation test of 110–140 ppm ClO_2_ gas for 6 h was performed in pigs [21]. By substituting the concentration corresponding to this result into our data, it corresponds to chamber #4 (Table 1). Since 10 to 19 ((4.13~7.85) × 10^5^/m^3^) Dr.CLO^TM^ sticks are required, it is usually not necessary to activate such a large amount of Dr.CLO^TM^ in a living space where ventilation is completely blocked. In the study of Akamatsu, when 0.1 ppm ClO_2_ gas was inhaled by rats for 24 h/day and seven days/week for six months, non-toxic results were obtained [16]. This is different from our study design because rats repeatedly inhaled ClO_2_ over a long time. However, it is a result that suggests the possibility that ClO_2_ gas can be non-toxic, even if it is inhaled for a long time at a sufficiently low concentration. However, since this result is an animal test result, it cannot be directly translated to humans. However, it can be expected that the concentration is sufficiently low at 0.1 ppm or below because there are many cases where even people do not recognize the smell, according to EPA reports [34,57,58]. In our experimental results, it is considered that the morphological characteristics of the mice lung tissues did not show any significant change compared to that of the control group within the concentration range of 20–50 ppm, so it is considered that the mice showed considerable resistance to exposure within a short period of time. The reaction principle of Dr.CLO^TM^ was briefly explained in Methods. It is specifically designed to generate ClO_2_ gas continuously for a long period of time in a certain concentration range (Figure 1) after consideration of safety and economy. It is very small and light, so it can be used in various indoor spaces. Since gas generation was maintained for about 42 days at a concentration of 0.011 ppm/m^3^ or higher than 0 ppm, it corresponds to a very much lower concentration than the dangerous concentrations reported in several previous studies [9,21,34,55,56,57]. Clearly, the results of this study are from animals, so direct application to humans may be complex. Furthermore, it is still necessary to obtain additional results through chronic repeated inhalation toxicity studies. Therefore, it would be a mistake to directly substitute these results into people with reference only to the results in this section.

According to several reports, ClO_2_ gas can kill viruses [7,9,17,18,23,43,59]. ClO_2_ has explicitly been known to exert its antiviral effects on viral nucleic acids, viral proteins [23,60] and oxidizing the amino acids tryptophan and tyrosine [17,23]. In particular, according to a recent study by Ogata and Miura (2020), ClO_2_ may inhibit the binding of the recombinant spike protein of SARS-CoV-2 to its receptor molecule, angiotensin-converting enzyme 2 (ACE-2) [59]. Hatanaka et al. (2021) concluded that ClO_2_ had a potent antiviral effect, even in the presence of organic matter [23]. However, unfortunately, due to the limited facilities that directly deal with coronavirus, adenovirus was used in this study instead. Since it is not easy to measure the effect of ClO_2_ gas by direct virus contact, the amount of reduced amplification of the infected virus in HBE cells was measured (Figure 2). The present study measured the inhibition of recombinant adenoviral amplification inoculated into HBE cells using the ICC and ELISA method. This is different from the typical method that measures the inhibition of virus proliferation by directly exposing cells to ClO_2_. The results Section 3.2 detected chloride in the non-treated group that was not treated with ClO_2_ gas. Since the chloride present in the medium is derived from chlorine dioxide, the absorbed chloride from the Dr.CLO^TM^ can be measured by subtracting the dissolved chloride present in the medium. For this reason, the experimental design was structured in this current study.

Therefore, the antiviral effect of chlorine dioxide gas seems to be exerted by attacking the common structures, regardless of the DNA or RNA of viruses. For this reason, ClO_2_ is expected to be effective against most viruses, and it is considered that the effect of ClO_2_ against the adenovirus used in this study was excellent. In particular, ClO_2_ gas is several times more potent than sodium hypochlorite, and it is known that it will exert its effect in various environments. EPA-certified product ingredients against coronavirus in the U.S. mostly function by artificial direct contact with the virus [10]. Since gas-type sterilizers have an easy and convenient sterilization effect, even in hard-to-reach areas, chlorine dioxide gas-type disinfectants are expected to receive more attention in the future. The critical core advantage of Dr.CLO^TM^ is that it can release ClO_2_ gas at a constant level in a safe concentration for evaporation (Figure 1). The results of this study indicate that it can work effectively to disinfect the surrounding environment without affecting human tissues. The maximum concentration from one Dr.CLO^TM^ stick was 0.011 ppm/m^3^. The concentration that could be irritating to humans was reported to be much higher than this [20,21,35,57,58,59], so in general, 1~3 Dr.CLO^TM^ sticks in an indoor space (more than 1 m^3^) where people live would not be expected to cause problems in a healthy person.

Since it is difficult to evaluate the toxicity of ClO_2_ gas in humans directly, the toxicity of chlorine dioxide gas was measured indirectly in the lungs using experimental animals. According to Young’s review, as a safe disinfectant, ClO_2_ has an inhibitory effect on bacteria, viruses, and fungi. It has been recognized as an effective substance with very little toxicity [12]. In our study, a single-dose inhalation toxicity test was conducted according to the guidelines of the ‘Single dose inhalation toxicity study (Appendix 10)’ in the ‘Standard for Toxicity Study of Pharmaceuticals’ provided by the MFDS [31] by applying a high ClO_2_ gas concentration to mice that could not be realistically reached in daily life. Of course, it was impossible to fix a specific concentration due to the material characteristics of chlorine dioxide gas itself. However, to obtain an approximate LC_50_ (approximately 68 ppm for males and approximately 141 ppm for females) by exposure to chlorine dioxide gas, inhalation toxicity evaluation had to be conducted using such high concentrations of ClO_2_ gas environment in the present study. Although several studies on chlorine dioxide have been conducted and the safety evaluations of various conditions have been conducted [12,61,62]; in most studies, like our results, significant changes in survival rate (Figure 3 and Table 3) and histopathology (Figure 4 and Figure 5, Table 4, Table 5 and Table 6) were found at notably high ClO_2_ concentrations. A remarkable observation in our study was a gender-related response to chlorine dioxide gas (Figure 3, Figure 4 and Figure 5). According to our results, females had higher resistance to ClO_2_ gas than males. From a histopathological perspective, males were more sensitive to ClO_2_ gas concentration than females (Figure 3, Figure 4 and Figure 5). Various index scores related to lung function and the ratio of the area occupied by the alveolar cavity per unit area confirmed that the animal’s respiratory function performance was typical, even under conditions of a relatively high ClO_2_ concentration. However, the normal range for females was higher than that in males (Figure 3, Figure 4 and Figure 5, and Table 3, Table 4 and Table 5). Therefore, it is thought that the effect of ClO_2_ gas generated by one Dr.CLO^TM^ stick on living organisms is insignificant.

The most challenging part of the experiment was the variation in the ClO_2_ gas concentration. Since ClO_2_ is decomposed by itself due to various causes, it is necessary to maintain the same indoor environment (illuminance, experimenter movement, etc.) as well as the ventilation water in the chamber.

One drawback of our animal experiments is that larger animals were not chosen. According to the ‘OECD Guidelines for the Testing of Chemicals’, healthy young adult rats are the preferred animal model [63]. If rats could be used in the inhalation toxicity test, relatively more meaningful results could have been obtained than when mice were used. However, it was challenging to perform in our experimental environment when using rats because more chambers required several powerful ventilating devices. Moreover, the MFDS guidelines specify that mouse selection is possible [31].

Although it was not possible to include all the information on ClO_2_ gas here, it was found that there is a reasonable possibility that it can be used safely in daily life and quarantine areas with the proper practical control. The use of ClO_2_ gas could be beneficial to combat the current COVID-19 pandemic situation and the usefulness of the newly designed Dr.CLO^TM^ could be further studied for safety and economy. Based on the results of this study, the improved control of ClO_2_ gas by Dr.CLO^TM^ should be confirmed through additional chronic repeated inhalation toxicity tests.

## 5. Conclusions

This study confirmed that the use of ClO_2_ gas adjusted by Dr.CLO^TM^ effectively inhibited adenovirus amplification in HBE cells and resulted in some histopathological changes in the respiratory system of ICR mice at a slightly higher concentration of 20–50 ppm. However, when looking at the functional aspects of the lungs, they were not considered to be significantly different from usual. As for the survival rate, this was found to be higher in females than males, indicating gender differences. Through the results of this study, we will study effective ClO_2_ gas use by screening the risk factors that ClO_2_ may have through dose setting and blood analysis of ClO_2_ gas. The use of Dr.CLO^TM^ can be an effective way to perform indoor quarantine for various infectious germs safely that are currently prevalent. As a result, the authors are confident that this study can provide helpful results related to the choice of disinfectants used to combat COVID-19 pandemic.

## Figures and Tables

**Table 1:** Group composition by ClO_2_ gas concentration, real-time concentration of ClO_2_ gas, number of applied Dr.CLO^TM^ sticks, and conversion value of ClO_2_ gas concentration per cubic meter.

Group (M & F)	Real-Time ClO_2_ Concentration (ppm)	Dr.CLO^TM^ Stick Numbers	Conversion of Stick Number/m^3^
Control	not treated	0	0
Chamber #1 (*n* = 10)	240<	35~37	≒ (1.45~1.53) × 10^6^
Chamber #2 (*n* = 10)	200~240	21~36	≒ (8.68~14.88) × 10^5^
Chamber #3 (*n* = 10)	150~200	18~20	≒ (7.44~8.27) × 10^5^
Chamber #4 (*n* = 10)	100~150	10~19	≒ (4.13~7.85) × 10^5^
Chamber #5 (*n* = 10)	50~100	5~11	≒ (2.07~4.55) × 10^5^
Chamber #6 (*n* = 10)	20~50	2~6	≒ (8.27~24.80) × 10^4^

**Table 2:** Histopathological examination (xcoring).

Histopathological Examination of Lung	
Alveolar cell edema/Intra-alveolar infiltration/Congestion/ Hemorrhage/Alveolar wall disruption	Scale from 0 to 3
	0 = absence of pathology (<5% maximum pathology)
	1 = mild (<10%)
	2 = moderate (15–20%)
	3 = severe (20–25%)
Leukocyte infiltration	Severity of inflammation resulting from contusions
	0 = no extravascular leukocytes
	1 = <10 leukocytes
	2 = 10~45 leukocytes
	3 = 45< leukocytes

**Table 3:** Number of dead animals following high concentrations of single ClO_2_ gas inhalation.

M	D1	D2	D3	D4	D5	D6	D7	D8	D9	D10	D11	D12	D13	D14	Total
NT	0	0	0	0	0	0	0	0	0	0	0	0	0	0	0
*C-#1	10	0	0	0	0	0	0	0	0	0	0	0	0	0	10
C-#2	6	4	0	0	0	0	0	0	0	0	0	0	0	0	10
C-#3	8	2	0	0	0	0	0	0	0	0	0	0	0	0	10
C-#4	3	6	1	0	0	0	0	0	0	0	0	0	0	0	10
C-#5	1	3	0	0	0	0	0	0	0	0	0	0	0	0	4
C-#6	2	0	0	0	0	0	0	0	0	0	0	0	0	0	2
F															
NT	0	0	0	0	0	0	0	0	0	0	0	0	0	0	0
C-#1	10	0	0	0	0	0	0	0	0	0	0	0	0	0	10
C-#2	3	6	0	0	0	0	0	0	0	0	0	0	0	0	9
C-#3	3	3	0	0	0	0	0	0	0	0	0	0	0	0	9
C-#4	0	1	1	0	0	0	0	0	0	0	0	0	0	0	2
C-#5	1	1	0	0	0	0	0	0	0	0	0	0	0	0	2
C-#6	0	0	0	0	0	0	0	0	0	0	0	0	0	0	0

*C-#: Chamber number according to the distribution of ClO_2_ concentration by Dr.CLO^TM^; M = Male mice; F = Female mice; NT = Non-treated group.

**Table 4:** Statistics of various indices based on histopathological evaluation of the lungs from male mice.

M	Alveolar Edema	Intra-Alveolar Infiltration	Congestion	Alveolar Hemorrhage	Disruption	Total Mean Score	Leukocyte Infiltration
NT	0.30 ± 0.153	0.00 ± 0.000	0.00 ± 0.000	0.00 ± 0.000	0.00 ± 0.000	0.06 ± 0.060	0.60 ± 0.306
C-#1	1.44 ± 0.242 ^b,i^	1.89 ± 0.200 ^a,e^	2.00 ± 0.289 ^a,i^	1.67 ± 0.333 ^a,i^	1.11 ± 0.200 ^a^	1.62 ± 0.160 ^a^	2.44 ± 0.242 ^a,d^
C-#2	1.30 ± 0.213 ^b^	1.20 ± 0.133 ^a^	1.90 ± 0.233 ^a,g^	1.70 ± 0.260 ^a,e^	0.80 ± 0.249^a^	1.38 ± 0.193 ^c^	1.90 ± 0.277 ^a,e^
C-#3	1.17 ± 0.307 ^b^	1.33 ± 0.333 ^a^	1.00 ± 0.447 ^a,d^	1.17 ± 0.477 ^a,d^	1.50 ± 0.563 ^b^	1.23 ± 0.085 ^c^	1.50 ± 0.342 ^a^
C-#4	1.00 ± 0.149 ^b^	1.40 ± 0.163 ^a^	1.20 ± 0.249 ^a,h^	1.80 ± 0.291 ^b^	1.50 ± 0.269 ^b^	1.38 ± 0.136 ^c^	1.70 ± 0.213 ^b^
C-#5	1.90 ± 0.314 ^c^	2.10 ± 0.233 ^a^	1.70 ± 0.423 ^a^	2.30 ± 0.335 ^a^	2.10 ± 0.348 ^a^	2.02 ± 0.102 ^c^	2.40 ± 0.163 ^a^
C-#6	0.50 ± 0.189 ^a^	1.38 ± 0.183 ^a^	0.63 ± 0.263 ^c^	0.63 ± 0.263 ^a^	1.38 ± 0.324 ^a^	0.90 ± 0.196 ^c^	1.50 ± 0.189 ^b^

^a^, *p <* 0.0005; ^b^, *p <* 0.005; ^c^*, p <* 0.05 vs. NT. ^d^, *p <* 0.005; ^e^, *p <* 0.01 vs. cage 5. ^g^, *p <* 0.005; ^h^, *p <* 0.01 and ^i^, *p < 0.05* vs. cage 6. C-#: Chamber number according to the distribution of ClO_2_ concentration by Dr.CLO^TM^; M = Male mice; NT = Non-treated group.

**Table 5:** Statistics of various indices based on histopathological evaluation of the lungs from female mice.

F	Alveolar Edema	Intra-Alveolar Infiltration	Congestion	Alveolar Hemorrhage	Disruption	Total Mean Score	Leukocyte Infiltration
NT	0.20 ± 0.133	0.00 ± 0.000	0.10 ± 0.100	0.00 ± 0.000	0.00 ± 0.000	0.06 ± 0.040	0.20 ± 0.133
C-#1	1.90 ± 0.314 ^b,f,h^	2.00 ± 0.258 ^b,f^	2.00 ± 0.258 ^a,h^	2.10 ± 0.233 ^a,f,h^	1.90 ± 0.233 ^a^	1.98 ± 0.037 ^c^	2.30 ± 0.153 ^a,d^
C-#2	1.56 ± 0.338 ^b^	1.56 ± 0.176 ^b^	2.22 ± 0.278 ^a,g^	2.67 ± 0.167 ^a,d,g^	1.33 ± 0.289 ^a^	1.88 ± 0.252 ^c^	2.33 ± 0.167 ^a,e^
C-#3	1.20 ± 0.200 ^b^	1.40 ± 0.221 ^c^	1.90 ± 0.379 ^c^	2.40 ± 0.306 ^a,e,g^	1.30 ± 0.396 ^a^	1.64 ± 0.225 ^c^	2.00 ± 0.298 ^a^
C-#4	1.60 ± 0.245 ^b^	1.80 ± 0.200 ^a^	1.40 ± 0.600 ^a^	1.80 ± 0.583 ^b^	2.00 ± 0.632 ^a^	1.72 ± 0.102 ^c^	1.80 ± 0.200 ^b^
C-#5	0.89 ± 0.261 ^c^	1.00 ± 0.167 ^b^	0.33 ± 0.236 ^b^	1.33 ± 0.236 ^a^	2.00 ± 0.333 ^a^	1.10 ± 0.277 ^c^	1.44 ± 0.176 ^a^
C-#6	0.80 ± 0.249	1.30 ± 0.260 ^c^	0.30 ± 0.213 ^c^	1.30 ± 0.153 ^a^	2.00 ± 0.258 ^a^	1.14 ± 0.284 ^c^	1.60 ± 0.340 ^b^

^a^, *p* < 0.0005; ^b^, *p* < 0.005; ^c^, *p* < 0.05 vs. NT. ^d^, *p* < 0.005; ^e^, *p* < 0.01; ^f^, *p* < 0.05 vs. cage 5. ^g^, *p* < 0.005; ^h^, *p* < 0.05 vs. cage 6. C-#: Chamber number according to the distribution of ClO_2_ concentration by Dr.CLO^TM^; F = Female mice; NT = non-treated group.

**Table 6:** Area percentage (%) of space occupied by the lumen of the alveolar sac per unit area.

Group (M)	Area Percentage (%)	Group (F)	Area Percentage (%)
Non-treated	58.17 ± 2.003	Non-treated	59.35 ± 2.451
Chamber #1	42.22 ± 3.411 ^a^	Chamber #1	44.24 ± 3.241 ^b^
Chamber #2	43.11 ± 1.791 ^a^	Chamber #2	45.14 ± 2.984 ^b^
Chamber #3	42.90 ± 1.835 ^a^	Chamber #3	45.55 ± 2.225 ^b^
Chamber #4	43.44 ± 2.495 ^a^	Chamber #4	48.25 ± 2.332 ^b^
Chamber #5	44.78 ± 3.427 ^c^	Chamber #5	52.44 ± 2.551
Chamber #6	52.40 ± 2.604	Chamber #6	55.32 ± 2.110

^a^, *p* < 0.0001; ^b^, *p* < 0.0005; ^c^, *p* < 0.005 vs. non-treated group.
